# A Distributed Multi-Agent Formation Control Method Based on Deep Q Learning

**DOI:** 10.3389/fnbot.2022.817168

**Published:** 2022-03-31

**Authors:** Nianhao Xie, Yunpeng Hu, Lei Chen

**Affiliations:** ^1^College of Aerospace Science and Engineering, National University of Defense Technology, Changsha, China; ^2^Hunan Key Laboratory of Intelligent Planning and Simulation for Aerospace Missions, Changsha, China; ^3^National Innovation Institute and Defense Technology, Beijing, China

**Keywords:** multi-agent system, distributed control, formation control, deep Q learning, collision avoidance

## Abstract

Distributed control method plays an important role in the formation of a multi-agent system (MAS), which is the prerequisite for an MAS to complete its missions. However, the lack of considering the collision risk between agents makes many distributed formation control methods lose practicability. In this article, a distributed formation control method that takes collision avoidance into account is proposed. At first, the MAS formation control problem can be divided into pair-wise unit formation problems where each agent moves to the expected position and only needs to avoid one obstacle. Then, a deep Q network (DQN) is applied to model the agent's unit controller for this pair-wise unit formation. The DQN controller is trained by using reshaped reward function and prioritized experience replay. The agents in MAS formation share the same unit DQN controller but get different commands due to various observations. Finally, through the min-max fusion of value functions of the DQN controller, the agent can always respond to the most dangerous avoidance. In this way, we get an easy-to-train multi-agent collision avoidance formation control method. In the end, unit formation simulation and multi-agent formation simulation results are presented to verify our method.

## 1. Introduction

In recent years, with the development of manufacturing, microelectronics, and communication technology, unmanned multi-agent systems (MASs), such as unmanned land vehicles, unmanned underwater vehicles, and unmanned aerial vehicles have emerged. Taking the advantage of tireless, fearless, and infallible characters over a human being, MASs begin to be applied in many areas, e.g., express distribution, disaster search and rescue, ecological monitoring, entertainment performances, and military confrontation. As a primary guarantee for MAS coordination and cooperation during task execution, formation control has received more and more extensive attention. Although there are many theoretical achievements, lots of formation control methods for MAS still lack autonomy in practical applications, where manual remote control or trajectory planning is needed to coordinate the agents. This is the main reason that MAS cannot cope with many tasks that require high timelines.

Researchers usually turn the formation control problem into a consistency problem and model the topology among agents using the undirect (Eren et al., 2003) or direct (Falconi et al., 2011) graph. Based on the communication or observation topology, the stability and convergence of the designed formation control protocol can be proved. Nevertheless, this kind of method (Li et al., 2019a; Guo et al., 2020) takes the agents as a mass point and neglects their volume, causing these methods insecure for possible collision between agents. In addition, the obstacles in the environment are usually indescribable, which also raises challenges for these methods. The potential function is widely used to describe obstacles. Using leader-follower topology, Liang et al. (2020) came up with an adaptive leader-follower formation control method for unmanned aerial vehicle (UAV) swarms with motion constraints and unknown disturbances, where the collision avoidance between UAVs is achieved with the artificial potential method. Merheb et al. (2016) modeled the environment as an incompressible flow field and designed a potential function for obstacles. Then panel method was applied to generate formation trajectory, i.e., streamlines of flow. Wu et al. (2016) proposed an obstacle envelope modeling method to model the obstacles. Each obstacle can be regarded as a dipole where the positive pole attracts agents and the negative pole distracts agents. However, trajectory planning methods require complex pre-design and calculation, making them only applicable in the mission planning stage and becoming invalid in on-board formation control. Behavior-based methods can also work to deal with obstacles. Xu et al. (2014) made behavior rules for agents to bypass obstacles and move along the walls. Lee and Chwa (2018) defined the inner, middle, and outer boundaries to wrap the obstacles so that agents can take effective collision avoidance behaviors in different boundaries. Although many details need to be considered, the behavior-based method cannot ensure stability and optimal during formation (Kamel et al., 2020).

To reduce reliance on the experience of engineers to make behavior rules, behavior learning methods begin to be applied in the formation control. Jin (2019) achieved stable tracking of followers to the leader with iteration learning method, where the only angle of sight observation is needed. Zhao et al. (2020) considered the relative distance constraints between agents and planned collision avoidance trajectory by iteration learning. Sanz et al. (2008) took the first step to apply the reinforcement learning method in the formation control. The agent with a *Q* learning controller can learn when to move forward and backward to keep aligned with the other two agents. However, when the state or/and action space become continuous, the corresponding Q table will be too large to describe or to train. The appearance of deep Q network (DQN) (Mnih et al., 2013) and deep deterministic policy gradient (DDPG) (Lillicrap et al., 2016) have solved this problem because continuous state and/or action space can be modeled by a neural network with limited weights. Sui et al. (2019) built long short-term memory (LSTM) networks to learn the formation controller of a follower to track the leader. The training is divided into two-stage. First, the network is supervised to learn the trajectory from the optimal reciprocal collision avoidance (ORCA) method (Van Den Berg et al., 2011), which is a well-known formation control method to deal with collision avoidance. Then, the agent explores better control protocol using reinforcement learning. Wang (2019) equipped the DDPG with double prioritized experience replay. Without considering collision avoidance, the command of roll angle for a UAV is generated by the DDPG controller and executed by a traditional PID controller. Although trained in the simulation environment, the learned roll angle command also works on hardware-in-the-loop simulation. However, Sui et al. (2019) and Wang (2019) only focus on the situation of one leader with one follower. Li et al. (2019b) trained multi-agent collision avoidance controller under decomposition methodology. At first, they predicted the value function from one-to-one collision avoidance rules using the iterative policy evaluation method. Then, the one-to-one value functions of multi-agent are fused and corrected to a multi-agent collision avoidance policy.

In this article, based on the decomposition methodology, we train a DQN for formation with leader-follower topology. First, we extract the simplest environment from the multi-agent formation control environment, i.e., one agent tracks its follower and needs only to avoid one obstacle. Then, in this simplest environment, the agent with DQN controller is trained with reshaped reward function and prioritized experience replay. Finally, through the min-max fusion of the DQN value functions, the agent can avoid more than one obstacle during formation control. The main contributions of this article are as follows:

- The multi-agent formation problem is decomposed to the pair-wise control problem, called the unit formation problem, which reduces the state dimension of DQN and thus, simplifies the learning of control policy.
- The reward function of the DQN controller is reshaped, which improves the training performance of DQN.
- By min-max fusion of DQN value function, the pair-wise controller is upgraded to a multi-agent formation controller.
- The action field is proposed to visually compare the DQN formation controller before and after reinforcement learning.

This article is organized as follows. In section 2, the multi-agent formation control problem is modeled. In section 3, after the proposed decomposition-fusion learning framework is sketched out, we explained the details of the unit formation controller, and a min-max fusion method to deal with multiple obstacles in multi-agent formation. In section 4, simulations are presented to validate our method. Finally, we conclude this article in section 5.

## 2. Problem Description

Oh et al. (2015) gave the general description of the formation control problem without considering collision avoidance, while, when considering the collision avoidance, the formation control problem can be modeled as follows. Supposed there are *N* agents in the formation, and let the state of agent *i* be **x**_*i*_ and the kinematics model and observation model are *f*_*i*_ and *g*_*i*_, respectively. The multi-agent state set is **X** = [**x**_1_, **x**_2_, ⋯ , **x**_*N*_], and the observation set is **Y** = [**y**_1_, **y**_2_, ⋯ , **y**_*N*_], and the control output set is **U** = [**u**_1_, **u**_2_, ⋯ , **u**_*N*_]. The target of multi-agent formation controller at time *t* is calculating control output set **U**_*t*_ according to states sequence **X**_*t*_0_:*t*_ and observations **Y**_*t*_0_:*t*_ from starting time *t*_0_ to current time *t* so that the agents can avoid collision with each other and form the expected geometric configuration. This problem can be described by optimization equations as follows.


(1) $$\{\begin{array}{l} \underset{U_{t_{0}:t}}{\min}\;\left\|F\left(X_{t}\right)-F\left(X^{*}\right)\right\|\\ \text{ s.t. }\;C(X)<0 \end{array}$$

where the function *F*(·) maps the states of agents to geometric constraints and the function *C*(·) is collision function. When a collision happens, *C*(**X**)≥0. The optimization objective is to make the geometric configuration *F*(**X**) converge to the expected *F*(X^*^). The states transformation and observation of agent *i* obey the following equation.


(2) $$\{\begin{array}{l} {\dot{x}}_{i}=f_{i}\left(x_{i},u_{i}\right)\\ y_{i}=g_{i}\left(X\right) \end{array}$$

## 3. Formation Control Method

In this section, the decomposition-fusion framework to train the formation controller is proposed. Then, the unit controller is designed and learned by the improved deep Q learning method to get a pair-wise policy. Finally, the min-max fusion method that makes the pair-wise policy applicable for multi-agent formation is elaborated.

### 3.1. The Decomposition-Fusion Framework

In a multi-agent formation, as the number of agents increases, each agent needs to communicate and cooperate with more agents, which require higher computation capacity. By designing a suitable formation topology, the relationship among agents can be simplified so that the communication and calculation burden is relieved.

**Figure 1:**
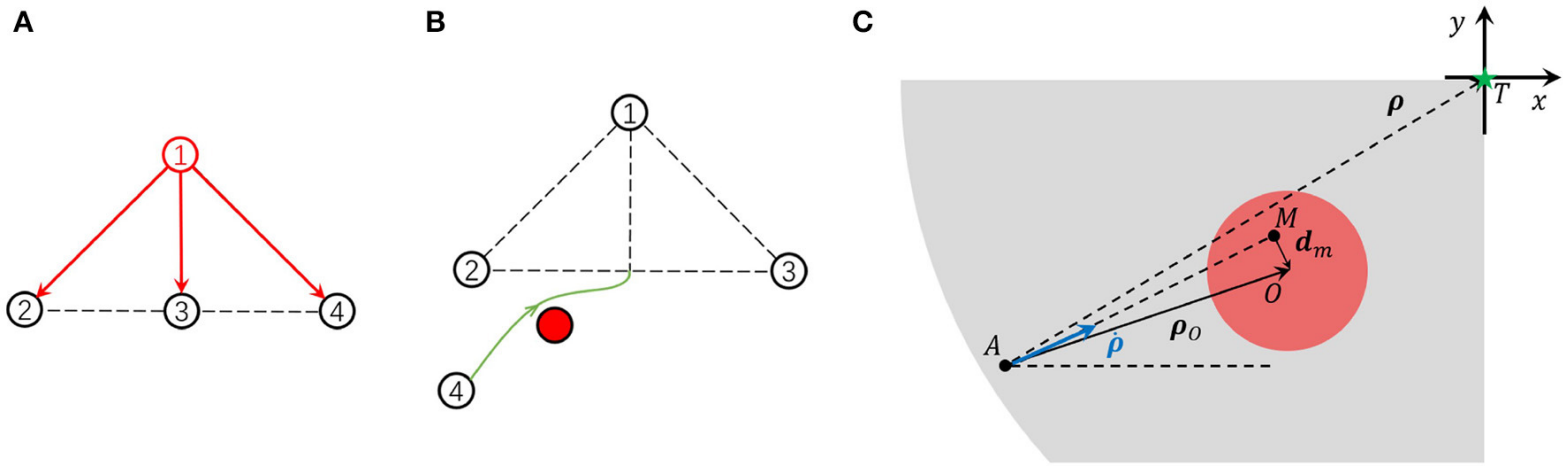
**(A)** The leader-follower topology in the formation control. **(B)** The unit problem of formation control. **(C)** The relative kinematics model.

With leader-follower topology, the formation can be automatically kept and globally controlled by the leader. In the clustered MAS, considering that the follower in one cluster can become the leader in other clusters, this kind of hierarchical topology makes the control of a large-scale system possible. As shown in Figure 1A, the follower calculates its expected relative position by observing its leader and then moves toward the destination. At the same time, the follower is not allowed to collide with the other agents in the formation. From the agents' point of view, an agent takes other agents in the formation as moving or static obstacles. The agent aims to observe the leader, move toward the relative destination, and meanwhile, avoid collision with those obstacles. Thus, the formation control problem can be treated as an obstacle avoidance problem from this insight. A formation controller is expected to avoid multiple obstacles. Instead of using a one-step learning framework that directly takes multiple obstacles into account, we proposed the two-step decomposition-fusion learning framework which can give the agent the ability to deal with multiple obstacles.

**Figure 2:**
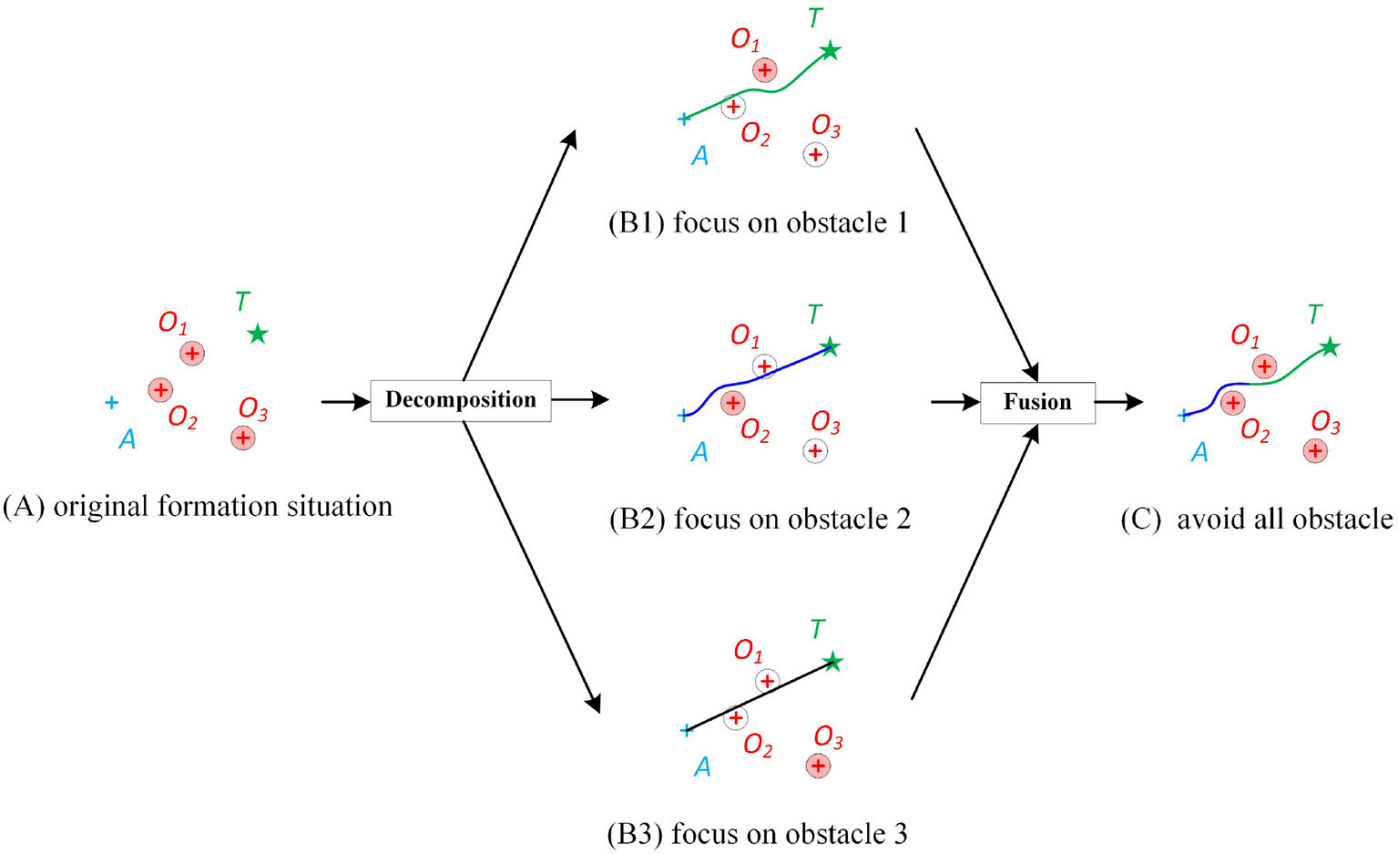
Diagram of the proposed decomposition-fusion formation control framework. **(A)** A formation situation that the agent *A* is supposed to destinate target *T* but may be blocked by multiple obstacles *O*_1_, *O*_2_, and *O*_3_. The circle of the obstacle represents its collision zone. If the trajectory of the agent does not intersect with the collision zone, it means that the agent has no collision with the obstacle. **(B)** The decomposition of original formation by focusing obstacles one by one. The “focused” obstacle is colored by red while the “ignored” obstacles are colored white. **(C)** The trajectory from the fused controller.

As shown in Figure 2A, assuming that there are three obstacles, a direct way is to learn a controller that takes the observations of all obstacles as input. But this leads to two troubles. One is that, as the number of obstacles increase the input dimension, learning samples, and the parameters increase, which increases the learning difficulty. The other one is that, if the number of obstacles is not three, e.g., two or four, the learned controller will be inapplicable.

In this article, a decomposition-fusion framework is proposed to solve the above problems. Inspired by the pair-wise policy (Kuchar and Yang, 2000) and attention mechanism (Mnih et al., 2014), in the decomposition stage, we assume that the agent only focuses on a certain obstacle, and was “blind” to the rest obstacles. Such a “uint controller” has a fixed input dimension and is relatively simple to learn. However, it is clear that the unit controller cannot ensure collision avoidance to all obstacles at once. As shown in Figure 2B1, when the agent pays attention to obstacle 1, the agent may not be able to avoid obstacle 2. Figures 2B2,B3 show similar things. Thus, a “fusion controller” will be designed to make the agent learn how to allocate attention and balance its pair-wise policy for different obstacles. In this way, an approximately global optimal solution can be gained.

### 3.2. Decomposition Stage: Reword Reshaped DQN for Unit Control

#### 3.2.1. Modeling of Unit Control Problem

In the uint problem, with the assumption that agents in *d*-dimension space have a second-order linear kinematics model, only the relative movement of agent *A*, agent's target position *T*, and obstacle *O* need to be considered. As shown in Figure 1C, *A, T, O* is the agent's current position, expected relative position, and obstacle center, respectively. The red circle is the threat zone and the gray zone is the motion permitted zone. **d**_*m*_ is the predicted minimum distance from the agent to the obstacle center if the agent keeps the current moving direction. We define a relative coordinate system in which the origin of the coordinates is fixed on the target position *T* and its axis is parallel to one inertial coordinate. Denote the relative position from the agent to the target as ***ρ*** and the relative position to the obstacle as ***ρ***_*O*_. The agent's velocity in the relative coordinates is $\overset{∙}{\rho }$. Then, the state of the agent *i* is ${\text{x}}_{i}={\left[\begin{array}{cc} \rho_{i}^{⊤} & {\overset{∙}{\rho }}_{i}^{⊤} \end{array}\right]}^{⊤}$. The kinematics model of the agent is


(3) $$\{\begin{array}{l} {\dot{x}}_{i}=Ax_{i}+Bu_{i}\\ y_{i}=Cx_{i} \end{array}\;\text{where}\;A\text{​ }=\text{ ​}\left[\text{​}\begin{array}{cc} 0 & 1\\ 0 & 0 \end{array}\text{​}\right]⊗I_{d},B\text{​ }=\text{ ​}\left[\text{​​}\begin{array}{c} 0\\ 1 \end{array}\text{​​}\right]⊗I_{d},\;C=I_{2n}$$

where ⊗ is Kronecker product and ${\text{u}}_{i}\in {\mathbb{R}}^{d}$ is control output which has constraint ${\text{u}}_{i}\in U$. The agent has velocity constraints ${\text{v}}_{i}\in V$. The safe distance between agents is *d*_safe_ which means the formation would fail if any distance between two agents was less than *d*_safe_. We also limit the agent to move inside a circle area with radius *D*. If the agent moves close enough to the target position, i.e., |***ρ***| ≤ *d*_*e*_, the unit problem is solved and *d*_*e*_ is called formation error.

#### 3.2.2. Buiding Markov Decision Process (MDP) for Unit Problem

The MDP is commonly used to describe continuous decision problems. An MDP can be defined by the tuple $M=<S,A,T_{r},R,\gamma >$, where S is state space, A is action space, *T*_*r*_ is state transition function, *R* is reward function, and γ is decay coefficient. A time *t*, the agent chooses action $a_{t}\in A$ using policy π based on state observation $s_{t}\in S$. Then, the state transits to *s*_*t*+1_ at time *t*+1, where the transition probability is *Pr*(*s*_*t*+1_|*s*_*t*_, *a*_*t*_) = *T*_*r*_(*s*_*t*_, *a*_*t*_, *s*_*t*+1_). Meanwhile, the agent gets reward *r*_*t*+1_ = *R*(*s*_*t*_, *a*_*t*_, *s*_*t*+1_). The goal of the continuous decision is finding the best policy π^*^ which maximizes the cumulative expected reward $\overset{\infty }{\underset{t=0}{\sum }}\gamma^{t}r_{t}$.

The state value function and state-action value function of MDP are briefly introduced for the convenience of explaining reward shaping and DQN training. Before training the policy of the unit formation MDP, the way of interaction between the agent and the designed environment needs to be decided. Then, the details of other elements of the unit formation MDP, including state, action, transition function, and reward function, are discussed.

State value function. The policy π:S×A→[0,1] gives the probability of choosing one action at the current state, and obviously, $\underset{a\in A}{\sum }\pi \left(s,a\right)=1$. The state value function of policy π at state *s* can be denoted as *V*^π^(*s*), which means no matter what policy the agent uses before the state *s*, if the agent always uses policy π from the state *s* to the end of the decision process, then the cumulative expected reward from state *s*_*t*_ to *s*_∞_ is *V*^π^(*s*).


(4) $$\begin{array}{r} V^{\pi }\left(s\right)=E_{\pi }\left[\overset{\infty }{\underset{k=0}{\sum }}\gamma^{k}r_{t+k+1}\;|\;s_{t}=s\right] \end{array}$$

State-action value function. The state value after action *a* is state-action value *Q*^π^(*s, a*), which is the cumulative expected reward from state *s*_*t*_ to *s*_∞_ is *V*^π^(*s*) when the agent transits to new state *s*′ after acting action *a* and keeps using policy π from the state *s*′ to the end of the decision process.


(5) $$\begin{array}{r} Q^{\pi }\left(s,a\right)=E_{\pi }\left[\overset{\infty }{\underset{k=0}{\sum }}\gamma^{k}r_{t+k+1}\;|\;s_{t}=s,a_{t}=a\right] \end{array}$$

**Figure 3:**
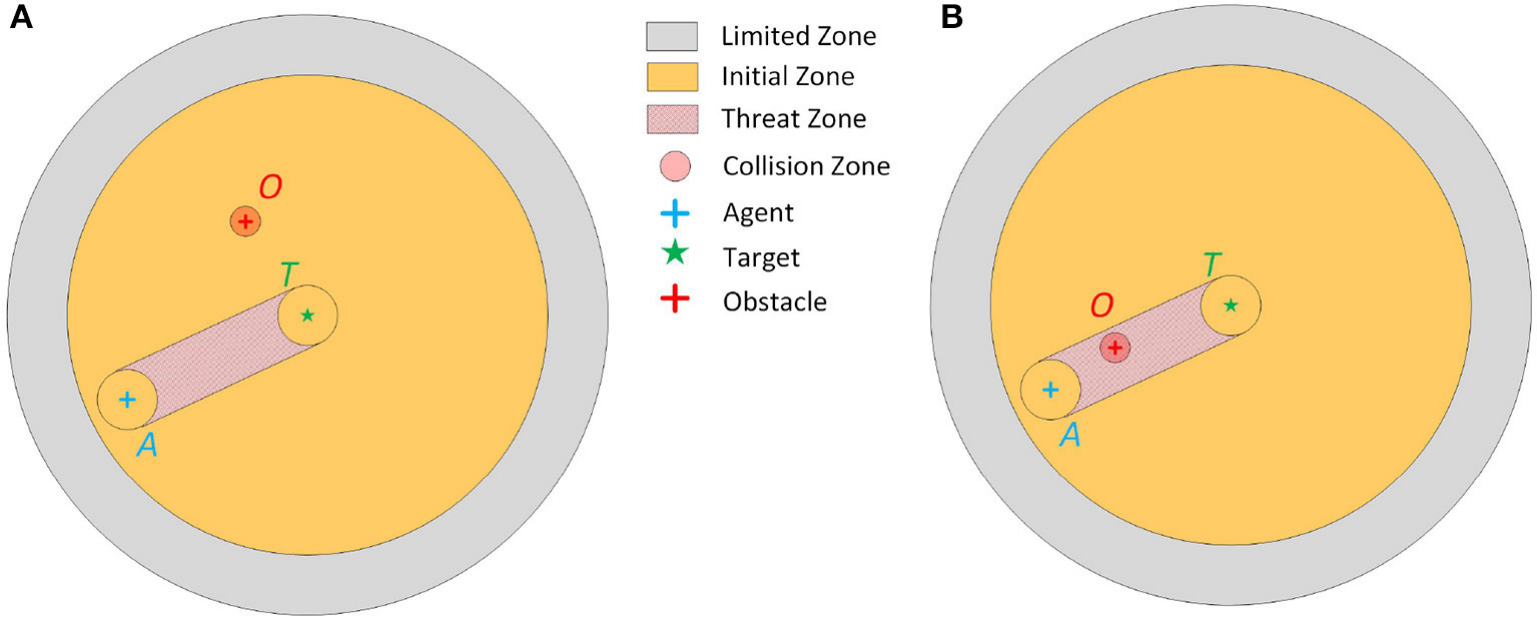
**(A)** The episode mechanism of unit formation problem when the obstacle O is out of the threat zone. **(B)** The episode mechanism of unit formation problem when the obstacle O is in the threat zone.

Episode mechanism. The samples of reinforcement learning are generated during the agent's exploration in the environment. Therefore, how the agent interacts with the environment needs to be decided, which is called episode mechanism in this article. Consider the environment in two-dimension space, in order to simulate the collision avoidance during formation control, we design the episode mechanism as illustrated in Figure 3. Let the target position be the center of the initial zone and limited zone, where the initial zone and limited zone are circular area with radius *d*_3_ and *D*, respectively. At the beginning of every episode, the agent is randomly initialized in the initial zone. A direct idea is to place the obstacle randomly on the limited zone. However, in most instances, the agent can simply move to the target along a straight line, which makes the agent lack of experience to learn how to avoid obstacle. Considering that an obstacle in the route of the agent to its target will threaten the agent, the threat zone is defined as an double-ended-wrench like area, where the width of the double-ended wrench is *d*_1_. The initialization strategy of the obstacle is as follows. In 50% of cases (as shown in Figure 3A), the obstacle is initialized randomly in the limited zone except threat zone, in the other 50% cases (as shown in Figure 3B), the obstacle is initialize randomly in the threat zone. The initial velocity of the agent is also random but the obstacle is assumed to be static for simplification. At each control time step, the agent receives command from the DQN controller and executes this action. This process keeps going until the following events occur:

- the agent reaches its target position (finish)
- the agent collides with the obstacle (collision)
- the agent moves outside the limited zone (out of range)
- the agent moves more than *n*_max_ step (out of step)

Therefore, the four kinds of state, i.e., finish, collision, out of range, and out of step are the terminal states of one episode.

MDP State. The unit formation control involves the agent, the obstacle, and the target. Therefore, the MDP State $s_{t}={\left[\rho^{⊤}\left(t\right),{\overset{∙}{\rho }}^{⊤}\left(t\right),\rho_{O}^{⊤}\left(t\right)\right]}^{⊤}={\left[x_{t},y_{t},v_{x,t},v_{y,t},x_{O,t},y_{O,t}\right]}^{⊤}$, i.e., the agent's relative position to target *x, y*, relative velocity *v*_*x*_, *v*_*y*_, and agent's relative position to the obstacle *x*_*O*_, *y*_*O*_ at step *t*, which contains enough information to calculate control output. In addition, the velocity constraint is *v*_*x*_, *v*_*y*_∈[−1, 1].

Transition function. The transition function of the MDP state is based on the agent's discrete kinematic Equation (3) but added the obstacle observation.


(6) $$\begin{array}{r} \left[\begin{array}{c} x_{t+1}\\ y_{t+1}\\ v_{x,t+1}\\ v_{y,t+1}\\ x_{O,t+1}\\ y_{O,t+1} \end{array}\right]=\left[\begin{array}{cccccc} 1 & 0 & dt & 0 & 0 & 0\\ 0 & 1 & 0 & dt & 0 & 0\\ 0 & 0 & 1 & 0 & 0 & 0\\ 0 & 0 & 0 & 1 & 0 & 0\\ 1 & 0 & dt & 0 & 0 & 0\\ 0 & 1 & 0 & dt & 0 & 0 \end{array}\right]\left[\begin{array}{c} x_{t}\\ y_{t}\\ v_{x,t}\\ v_{y,t}\\ x_{O,t}\\ y_{O,t} \end{array}\right]+\left[\begin{array}{cc} 0 & 0\\ 0 & 0\\ 1 & 0\\ 0 & 1\\ 0 & 0\\ 0 & 0 \end{array}\right]\left[\begin{array}{c} u_{x}\\ u_{y} \end{array}\right] \end{array}$$

where *u*_*x*_, *u*_*y*_ is the formation control command and *dt* is the time interval.

Action. The MDP action is directly defined to be the formation control command of the agent. Considering discrete action space, the action space is


$$\begin{array}{l} a=[u_{x},u_{y}]\in \{[0,0],[-2,0],[-1,0],[1,0],[2,0],[0,-2],\\ \left[0,-1],[0,1],[0,2]\right\} \end{array}$$

Reward function. As mentioned in the episode mechanism, one episode will be terminated under four situations, i.e. finish, collision, out of the range, and out of the step. Correspondingly, there are four kinds of terminal rewards for the unit formation MDP. Let the original reward function be:


(7) $$R(s)=\{\begin{array}{ll} \;2, & if\;finish\\ -2, & f\;collision\\ -2, & if\;out\;of\;range\\ \;0, & otherwise \end{array}$$

#### 3.2.3. Reward Shaping

Although the DQN can be trained by the original reward function Equation (8), the agent cannot get meaningful reward most of the time because the original reward is very sparse. Especially at the beginning of the training, it is hard for the agent to gain a way to the target. Therefore, the original reward function Equation (8) is not conducive to the convergence of training. In this article, we reshape the original reward to make the DQN get the reward at every step, which will improve the learning process.

For brevity, *s*_*t*_, *a*_*t*_, and *s*_*t*+1_ are abbreviated as *s, a*, and *s*′. Having original MDP M=<S,A,T,γ,R>, the reward-reshaped MDP can be denoted as $M^{\prime }=<S,A,T,\gamma ,R^{\prime }>$, where $R^{\prime }\left(s,a,s^{\prime }\right):S\times A\times S\to \mathbb{R}$ is reshaped reward


(8) $$\begin{array}{r} R^{\prime }\left(s,a,s^{\prime }\right)=R\left(s,a,s^{\prime }\right)+F\left(s,a,s^{\prime }\right), \end{array}$$

and $F\left(s,a,s^{\prime }\right):S\times A\times S\to \mathbb{R}$ is an additional reward that need to be designed to ensure that the optimal solution of the original MDP is the same as the reward-shaped MDP. According to the reward reshaping principle (Ng et al., 1999), if exits Φ(s):S→ℝ which makes *F*(*s, a, s*′) = γΦ(*s*′)−Φ(*s*), then the additional reward *F*(*s, a, s*′) is potential, which can ensure the invariance of optimal solution. Denote the state value function of the two equivalent MDP as $V_{M}^{\pi },V_{M^{\prime }}^{\pi }$ respectively, then


(9) $$\begin{array}{r} V_{M^{\prime }}^{\pi }=V_{M}^{\pi }-\Phi \left(s\right) \end{array}$$

If $\Phi \left(s\right)=V_{M}^{*}\left(s\right)$, then $V_{M^{\prime }}^{*}\left(s\right)\equiv 0$. Equation (10) theoretically indicates that the learning of $V_{M^{\prime }}^{*}\left(s\right)$ will be easier if we reshape the reward function by Φ(*s*) that predicts $V_{M}^{*}\left(s\right)$ (Ng et al., 1999). The agent has a higher state value when it approaches the target position, and the agent has a lower state value when the collision threats exist and the agent approaches the obstacle. Let


(10) $$\Phi (s)=\{\begin{array}{ll} -\rho & \text{ if }\rho_{O}>2d_{safe}\\ -\rho +(\rho_{O}-2d_{safe}) & \text{ if }\rho_{O}\;⩽\;2d_{safe} \end{array}$$

Finally, the additional reward function is defined as


(11) $$F(s,a,s^{\prime })=\{\begin{array}{ll} -\gamma \rho (s^{\prime })+\rho (s) & \text{ if }\rho_{O}>2d_{safe}\\ -\gamma \rho (s^{\prime })+\rho (s)+\gamma \rho_{O}(s^{\prime }) & \;\\ -\rho_{O}(s)+2(1-\gamma )d_{safe} & \text{ if }\rho_{O}\;⩽\;2d_{safe} \end{array}$$

#### 3.2.4. Q Learning for Optimal Policy

If the optimal state-action value function *Q* is known, the optimal policy is


(12) $$\begin{array}{r} \pi^{*}\left(s\right)=\underset{a}{\arg\max}Q^{*}\left(s,a\right) \end{array}$$

The Q learning (Sutton and Barto, 1998) can iteratively make the *Q* function approaches the optimal because the current state-action value function can be presented using the next state-action value function according to the Bellman equation, i.e.,


(13) $$Q^{\pi }(s,a)=\sum_{s^{\prime }}P_{ss^{\prime }}^{a}\left[R_{ss^{\prime }}^{a}+\gamma E_{\pi }[\sum_{k=0}^{\infty }\gamma^{k}r_{t+k+2}|s_{t+1}=s^{\prime }]\right]$$

where $P_{ss^{\prime }}^{a}=Pr\left(s_{t+1}=s^{\prime }|s_{t}=s,a_{t}=a\right)$ $R_{ss^{\prime }}^{a}=R\left(s_{t}=s,a_{t}=a,s_{t+1}=s^{\prime }\right)$. Therefore, the optimal state-action value function satisfies the equation


(14) $$\begin{array}{r} Q^{*}\left(s,a\right)=E_{s^{\prime }}\left[R\left(s,a,s^{\prime }\right)+\gamma \underset{a^{\prime }}{\max}Q^{*}\left(s^{\prime },a^{\prime }\right)\right] \end{array}$$

According to Equation (15), the *Q* function can be solved by temporal difference and eventually converge to Q^*^. In traditional *Q* learning method, the *Q* function is defined by numerical table, which is unsuitable when the state space becomes larger or even infinite. Mnih et al. (2013) used a deep network to model the *Q* table so that it is possible to define infinite states and actions with finite weights of the network. They built two networks called evaluation network *Q* and target network *Q*^−^, respectively. The structure of the two networks is the same, but they have different parameters. Denoting the parameter of evaluation network and target network as *w* and *w*^−^ respectively, the error of evaluation network to target network is


(15) $$\begin{array}{r} J\left(w\right)=E_{s^{\prime }}\left[{\left(R_{ss^{\prime }}^{a}+\gamma \underset{a^{\prime }}{\max}Q^{-}\left(s^{\prime },a^{\prime }\right)-Q\left(s,a\right)\right)}^{2}\right] \end{array}$$

the parameters of evaluation network can be updated by


(16) $$\begin{array}{r} w\leftarrow w+\alpha \nabla J=w+\alpha \left(R_{ss^{\prime }}^{a}+\gamma \underset{a^{\prime }}{\max}Q{\left(s^{\prime },a^{\prime }\right)}^{-}-Q\left(s,a\right)\right)\\ \nabla Q\left(s,a\right) \end{array}$$

where α is the learning rate. The parameters of target network *w*^−^ are updated to parameters of the evaluation network *w* every *N*_replace_ training step, making the parameters of evaluation networks approach the optimal parameters w^*^. In this way, the iterative temporal difference method is accomplished in DQN training.

**Figure 4:**
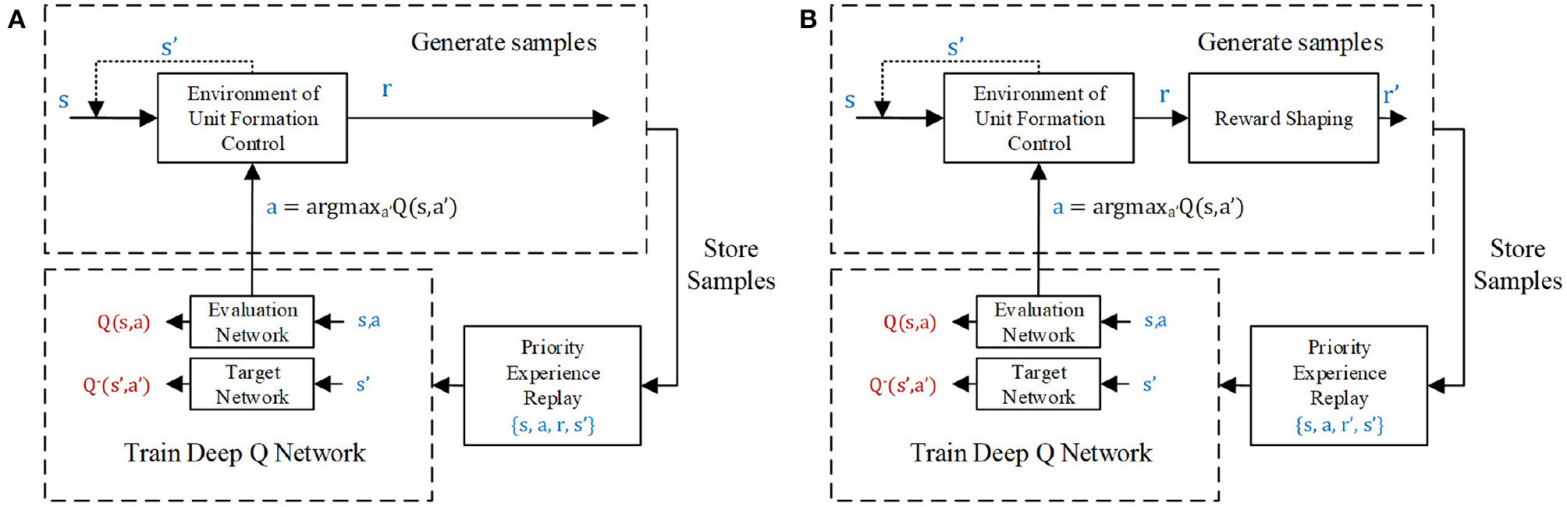
The unit formation deep Q network (DQN) controller learning process **(A)** without and **(B)** with reward shaping.

The training processes of unit formation problems with and without reward shaping are shown in Figures 4A,B, respectively. The training samples come from the experience (*s, a, r, s*′) of the agent, obtained by interacting with the environment. These samples are temporarily stored in the experience pool with size *N*_pool_. However, at every training step, only *N*_batch_ samples will be trained during overpopulation of the output error between the evaluation network and target network. Therefore, the priority experience replay method (Schaul et al., 2016) is employed in this article to increase the probability of samples with large errors. The samples with a high error are more likely to be selected to train the networks, which can speed up the learning process.

### 3.3. Fusion Stage: Multi-Agent Formation Control by Min-Max Fusion of Unit Formation Control Policy

The unit formation DQN controller only equips the agent with the ability to avoid one certain obstacle during formation. However, there will be more than one potential threat in the multi-agent formation control. To make the agent knows which obstacle needs to be preferentially treated with, the min-max fusion method proposed by Chryssanthacopoulos and Kochenderfer (2011) is employed to fuse pair-wise unit formation control policy.

**Figure 5:**
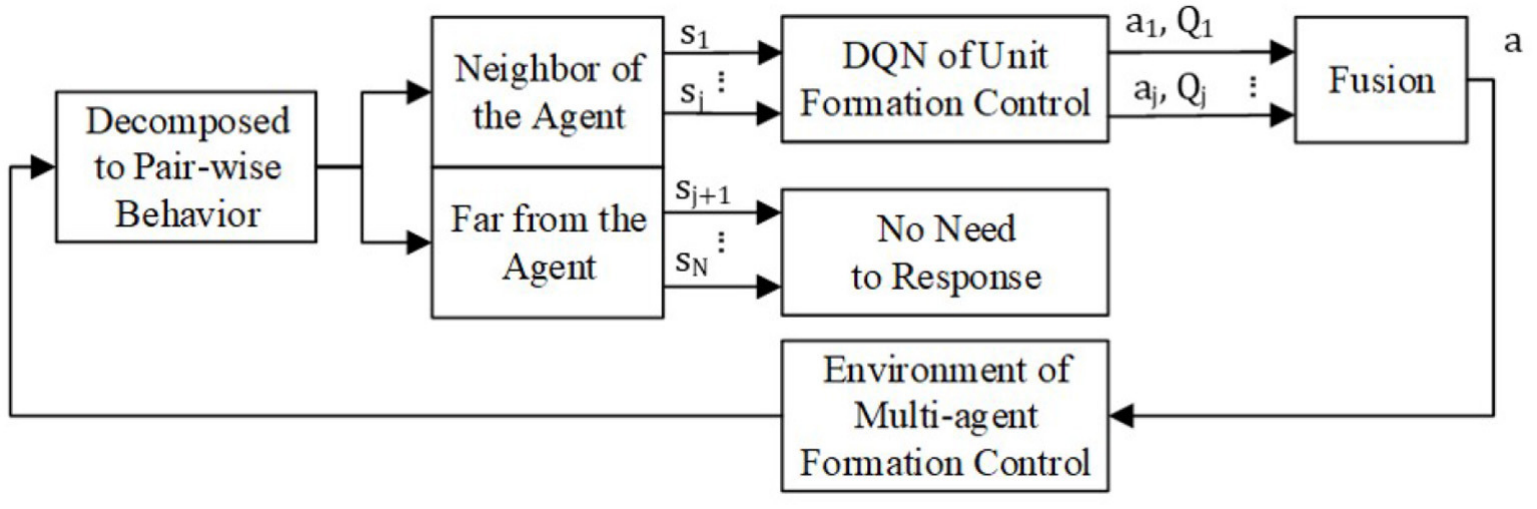
Policy fusion for multi-agent formation control.

To simplified the denotation, we omit the subscript *i*. The min-max fusion process is shown in Figure 5. The agent views other agent *j* in the formation as an obstacle. If the distance between agent *i* and *j* is beyond the threshold which makes the observation or communication impossible or the agent *j* is too far to threaten agent *i*, there is no need for agent *i* to respond to agent *j*. If not, having the state of agent *i* and *j* as input, the pair-wise policy can output the optimal action *a*_*j*_ and responding state-action function *Q*_*j*_. From the definition of the state-action value function in section 3.2.2, *Q*_*j*_ predicts the cumulative expected reward after executing action *a*_*j*_. A higher state-action value means lower collision threats. Thus, the lowest state-action value of all the optimal pair-wise policies most likely comes from the biggest threat. The min-max fusion method makes the agent respond first to the biggest threat. Therefore, the balanced global policy from the pair-wise policy is


(17) $$\begin{array}{r} a=\arg\underset{a_{j}\in A_{j}}{\min}Q\left(s_{j},a_{j}\right) \end{array}$$

and


(18) $$\begin{array}{r} a_{j}=\arg\underset{a^{\prime }\in A}{\max}Q\left(s_{j},a^{\prime }\right) \end{array}$$

where $A_{j}=\left\{a_{j}\right\}$ is all the pair-wise policy of the agent *i* to the other agents *j, j* = 1, 2, ⋯ , *N, j*≠*i*.

For every agent in the formation, it can get a global formation control policy without extra training by using Equations (18) and (19).

## 4. Simulations and Results

To verify our multi-agent formation algorithm step by step, we first present two demos of unit formation control in section 4.1. Then, in section 4.2, two more demos of multi-agent control are given to validate our method of multi-agent formation with collision avoidance.

### 4.1. Unit Formation Control Policy

#### 4.1.1. Training

**Figure 6:**
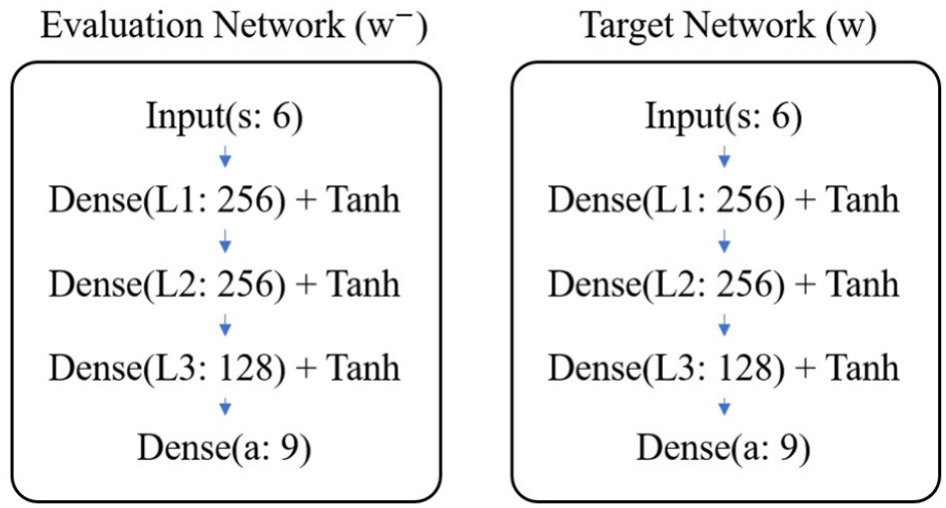
Structure of DQN.

As shown in Figure 6, the evaluation network and target network are both composed of three fully connected layers. There are 256, 256, and 128 neurons in the first, second, and third layers, respectively. The weights are initialized using Gaussian random *N*(0, 0.3^2^) and the biases are initialized using uniform random *U*(0, 0.1). Except for the last layer, the other layers' output is activated by tanh function.

The reward decay coefficient is set as 0.95 and the size of the experience pool to store samples is set as 50,000. For every *N*_replace_ = 2, 000 training step, the parameters of the target network will be replaced by those of the evaluation network. At the beginning of training, the ϵ-greedy probability is 0.95 which allows the agent to explore the environment as far as possible. As the training goes on, the ϵ linearly decreases by 0.01 every 100 episodes to limit the exploration range of the agent until reaches the minimum value ϵ_min_ = 0. The weights are updated by Adam Optimizer with an initial learning rate α = 10^−3^. Like the probability of ϵ-greedy exploration, the learning rate also decreases every 100 episodes, not linearly but exponentially, i.e., the learning rate becomes 0.99 times the old learning rate (α←0.99α). At every training step, *N*_batch_ = 32 samples are selected by the priority experience replay method. The training stops when the number of trained episodes reaches 20,000.

We assume that the episode is finished if the distance between the agent and its target position is less than *d*_*e*_ = 3, and the agent is safe if the distance between the agent and the obstacle is more than *d*_safe_ = 5. To generate samples, the inner and outer radius of the obstacle zone are *d*_1_ = 10 and *d*_2_ = 25, respectively. The agent is limited to moving within the circular zone (radius *D* = 100) around the target position. If the episode goes more than *n*_max_ = 100 control steps, or collision or crossing happens, the episode is forced to stop.

All the parameters related to the training of DQN is listed in Table 1.

**Table 1:** Parameter of deep Q network (DQN) and training.

**Parameters**	**Value**	**Parameters**	**Value**
Reward decay coefficient γ	0.95	update target network every *N*_replace_*step*	2 × 10^3^
Initial ϵ-greedy probability ϵ	0.95	Minimum ϵ-greedy probability ϵ_min_	0
Initial learning rate α	10^3^	Size of experience pool*N*_pool_	5 × 10^4^
Total episode *N*_*e*_	2 × 10^4^	Batch size *N*_batch_	32
Maximum step in each episode *n*_max_	100	Formation error *d*_*e*_	3
Safe distance *d*_safe_	5	Width of threat zone *d*_1_	10
Radius of limited zone *D*	100	Radius of initial zone *d*_3_	50
Simulation interval *dT*	0.1	Control interval *dT*_*c*_	1

To test the reward shaping in this article, we trained the original DQN and reward shaping DQN five times using the same episode mechanism, parameters, and network structure, but different network initial parameters, and different random seeds to initialize the agent's position, agent's velocity, and obstacle's position. During the training process, the DQN with and without reward shaping is tested. Let ${\bar{R}}_{\text{test}}\left(m\right)$ be the average reward of *N*_test_ = 300 test episodes after training DQN by *m* training episodes. Denote the DQN trained by *m* training episodes as *m*th DQN, the average reward of the *m*th DQN in one training process is


(19) $$\begin{array}{r} {\bar{R}}_{\text{test}}\left(m\right)=\overset{N_{\text{test}}}{\underset{k=1}{\sum }}\overset{n_{k}}{\underset{j=j_{0,k}}{\sum }}r_{k,j}\left(m\right) \end{array}$$

where *r*_*k, j*_(*m*) is the reward of the *j*th step in the *k*th test episode obtained by *m*th DQN. In addition, *j*_0, *k*_ = max{1, *n*_*k*_−10}, meaning that the average reward is the average of the last 10 steps when the total steps of *k*th episode are more than 10. To make the reward of DQN with and without reshaping comparable, the average reward is normalized by the maximum average reward during the whole training process.


(20) $$\begin{array}{r} {\bar{R}}_{\text{test}}^{\prime }\left(m\right)=\frac{{\bar{R}}_{\text{test}}\left(m\right)-\min_{n}\left\{{\bar{R}}_{\text{test}}\left(n\right)\right\}}{\underset{n}{\max}\left\{{\bar{R}}_{\text{test}}\left(n\right)\right\}-\min_{n}\left\{{\bar{R}}_{\text{test}}\left(n\right)\right\}},n=1,\cdots \;,N_{e} \end{array}$$

**Figure 7:**
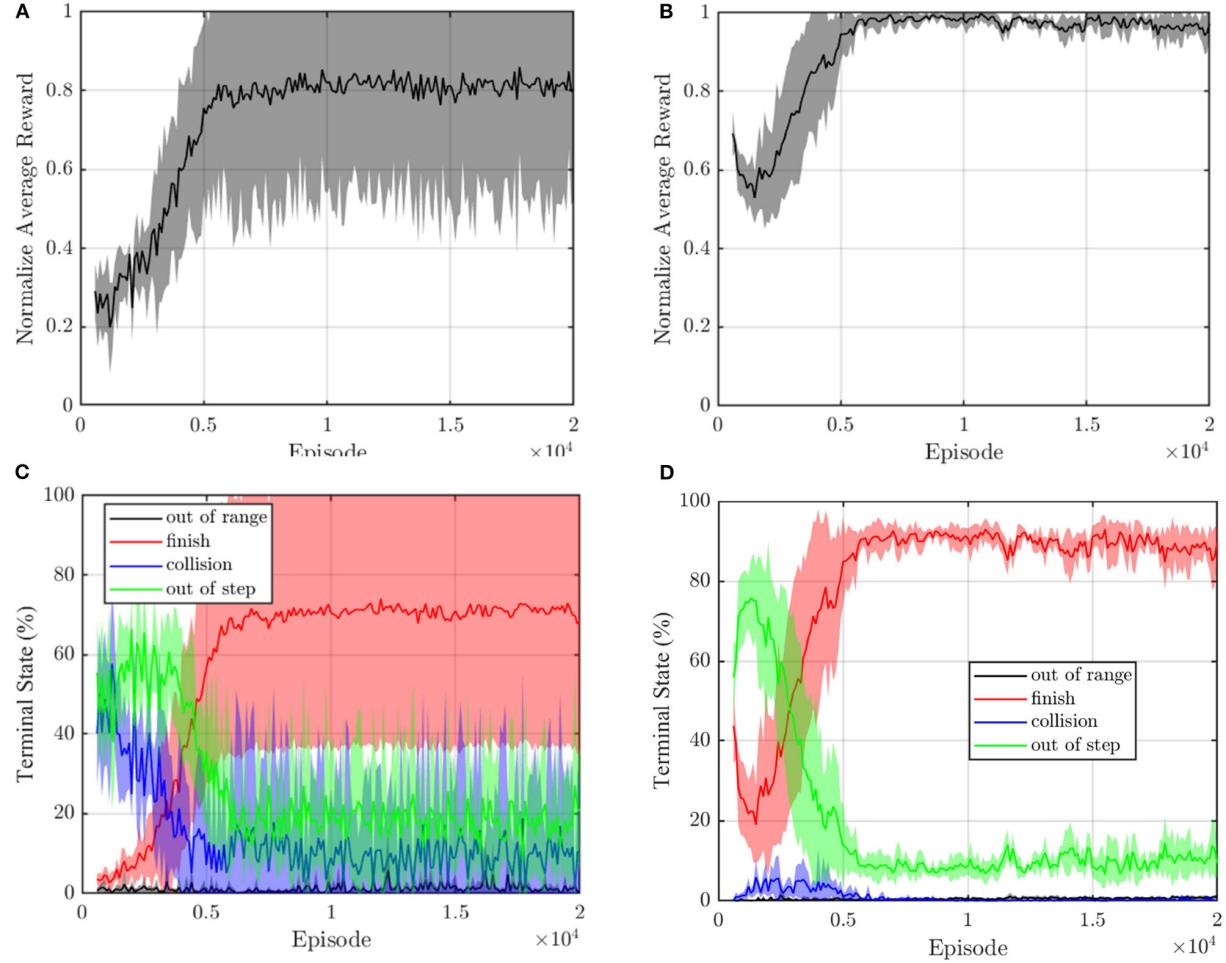
The normalized average reward of DQN **(A)** without and **(B)** with reward shaping. The terminal state during train process **(C)** without and **(D)** with reward reshaping. The translucent shadow their SE.

The mean curve and SE of the normalized average reward with and without reshaping are recorded in Figures 7A,B. As shown in Figure 7A, the normalized average reward without reshaping reaches the maximum at about 5,000 training episodes by 0.8 ± 0.2. In Figure 7B, the normalized average reward with reshaping grows to the maximum value at about 5,000 training episodes by about 0.95 and the SE is small. Therefore, the convergence process with reward shaping is more stable.

Figures 7C,D present the terminal states of test episodes. We call the episodes out of step, out of range, and collision as unfinished episodes. Both terminal state curves show a rising trend of the finished episodes. As shown in Figure 7C, without reward shaping, most episodes terminate due to the state of out of step, and collision before 5,000 episodes because the sparse reward makes it hard for the agent to get a positive experience. The finish rate end by about 75% and there still is a 10% collision probability. In Figure 7D, benefiting from the reward shaping, the failure episodes of the reward shaping controller are mainly out of range, and the collision episodes only occur before 5,000 steps which indicate that the agent effectively learns an obstacle avoidance strategy. The finish rate reaches about 90% with nearly zero collision probability. The reward shaping improves the convergence of terminal state curves. Therefore, we can conclude that the reward shaping method in this article improves the convergence of DQN.

#### 4.1.2. Demo: Visualized Action Field

**Figure 8:**
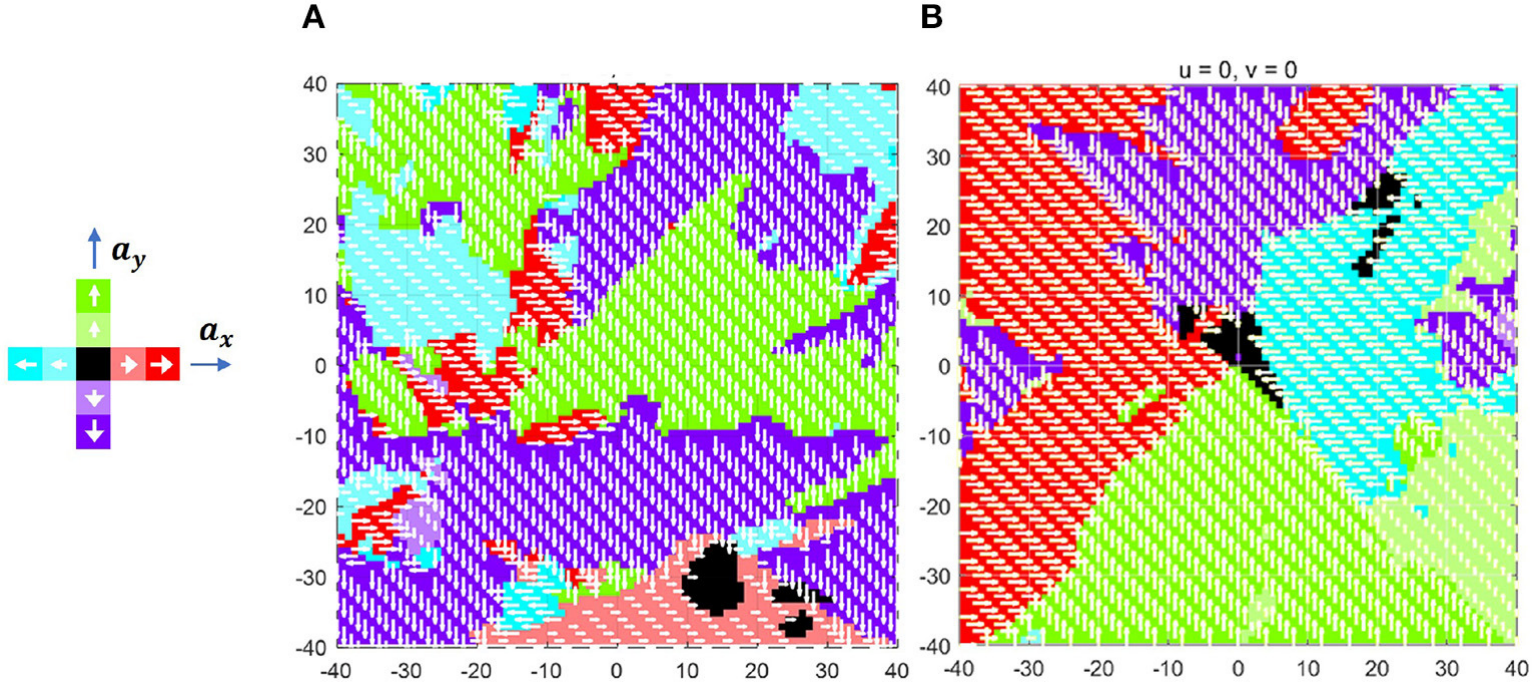
Action field **(A)** before and **(B)** after training.

Sui et al. (2019) colored the action space according to the probability that action is optimal to analyze the learned policy. However, the action space only shows the policy in some keyframes. To globally visualize the learned policy by DQN, the action field is defined as follows. Supposed that the current position and velocity of the agent is ***ρ*** and $\overset{∙}{\rho }$, the optimal action can be calculated by the DQN policy π. By fixing the velocity $\overset{∙}{\rho }$ but traversing the position ***ρ*** of the agent, the function mapping $F_{A}\left(\rho |\pi ,\overset{∙}{\rho }\right)$ represents the action field. In other words, the point in the action field is the optimal action when the agent of velocity $\overset{∙}{\rho }$ locates in the same position. Figure 8 shows the action field of $\overset{∙}{\rho }=0$ with the target position at point [0, 0] and limited zone in the square of [−40, 40]−−[−40, 40].

As noted, the green, red, purple, and blue colors represent up, right, down, and left action, respectively, in which deeper color indicates bigger acceleration. Since the parameters of DQN are randomly initialized before training, the zero-velocity action field at this time is chaotic, as shown in Figure 8A. After 20,000 episodes of training, as illustrated in Figure 8B, the action field becomes regular. More precisely, the action field is composed of four triangle zones, which make the agent always move toward the expected position.

#### 4.1.3. Demo: Unit Formation Control

**Figure 9:**
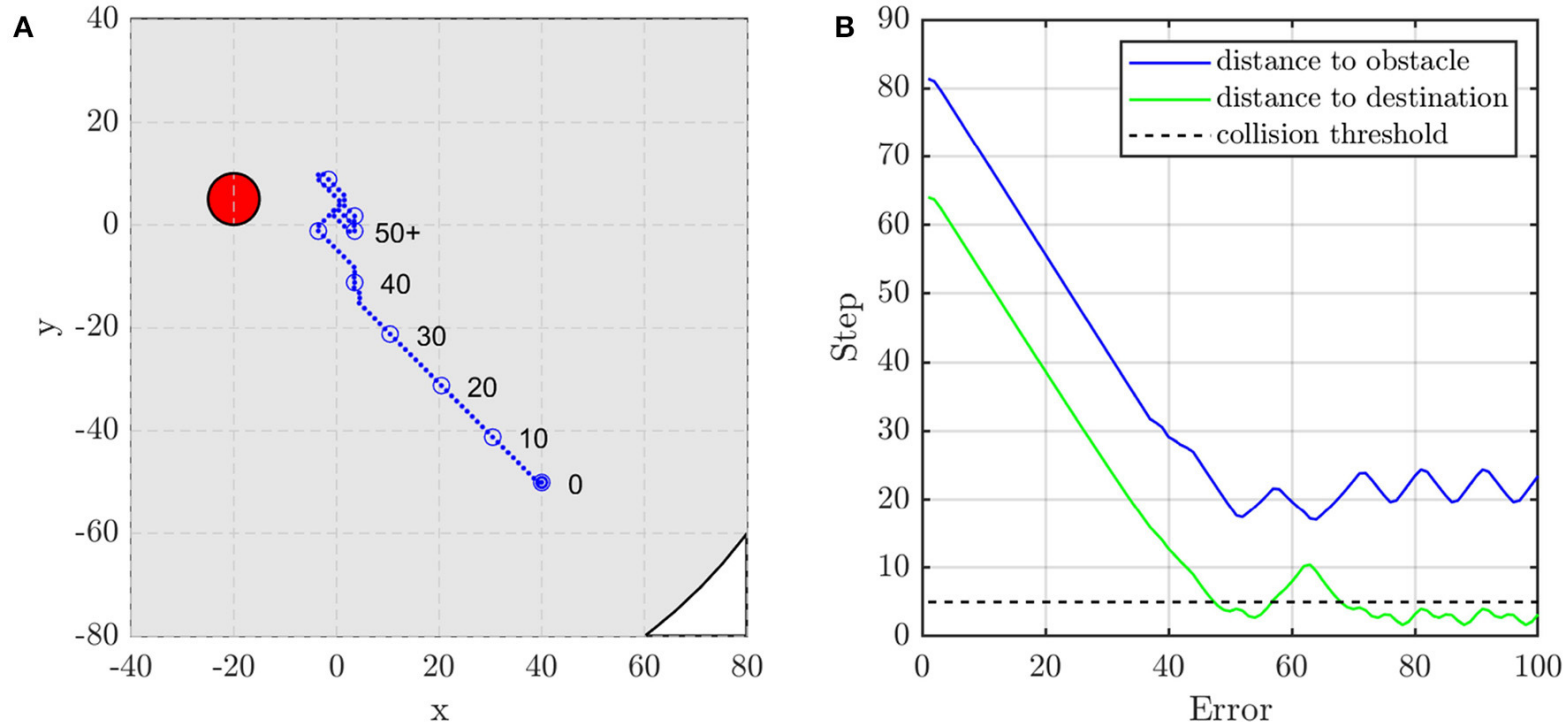
The agent's **(A)** trajectory, and **(B)** distance to obstacle and destination when the obstacle is not on the line between the agent's initial and target position. The red circle is the obstacle with the radius of *d*_*safe*_ and the blue dots are the trace of the agent.

**Figure 10:**
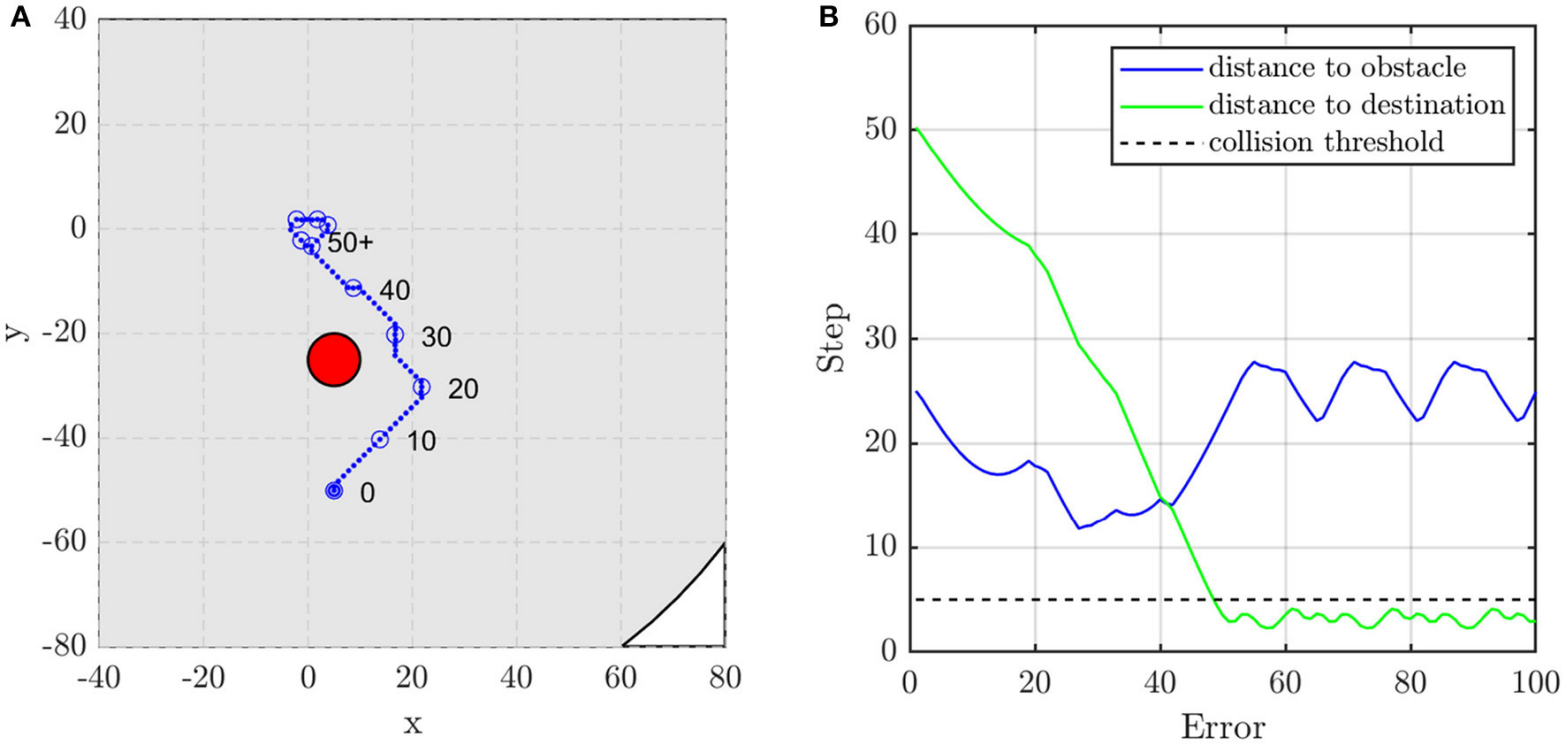
The agent's **(A)** trajectory and **(B)** distance to obstacle and destination when the obstacle is on the line between the agent's initial and target position. The red circle is the obstacle with the radius of *d*_*safe*_ and the blue dots are the trace of the agent.

In this subsection, two scenarios are presented to show the performance of the unit formation control. They represent two typical situations, i.e., the obstacle is or is not on the line between the initial position of the agent and the target position. When there is no obstacle in the direction to the target position, as shown in Figures 9A,B, the agent adjusts its direction at about the 15th and the 30th control step to aim at the target. If the obstacle blocks the way, the agent moves toward the target until the distance to the obstacle is close enough to alarm the agent. As shown in Figures 10A,B, the agent turns left at about the 22nd control step and turns right at about the 37th control step to avoid the obstacle.

Due to discrete acceleration and fixed time step, the zero-velocity constraint is not satisfied, when the agent arrives at the target position. This makes the trajectory of the agent fluctuate near the target position. It is noted that the agent does not need to avoid other agents when it is close enough to the target position. Therefore, to eliminate the continuously small fluctuation, in the following demos, the simple proportional derivative (PD) control method is employed when the agent is close enough to the target (assuming that the distance to the target is less than 10 in this article).

### 4.2. Multi-Agent Formation Control Policy

#### 4.2.1. Demo: Avoid Multiple Obstacles

**Figure 11:**
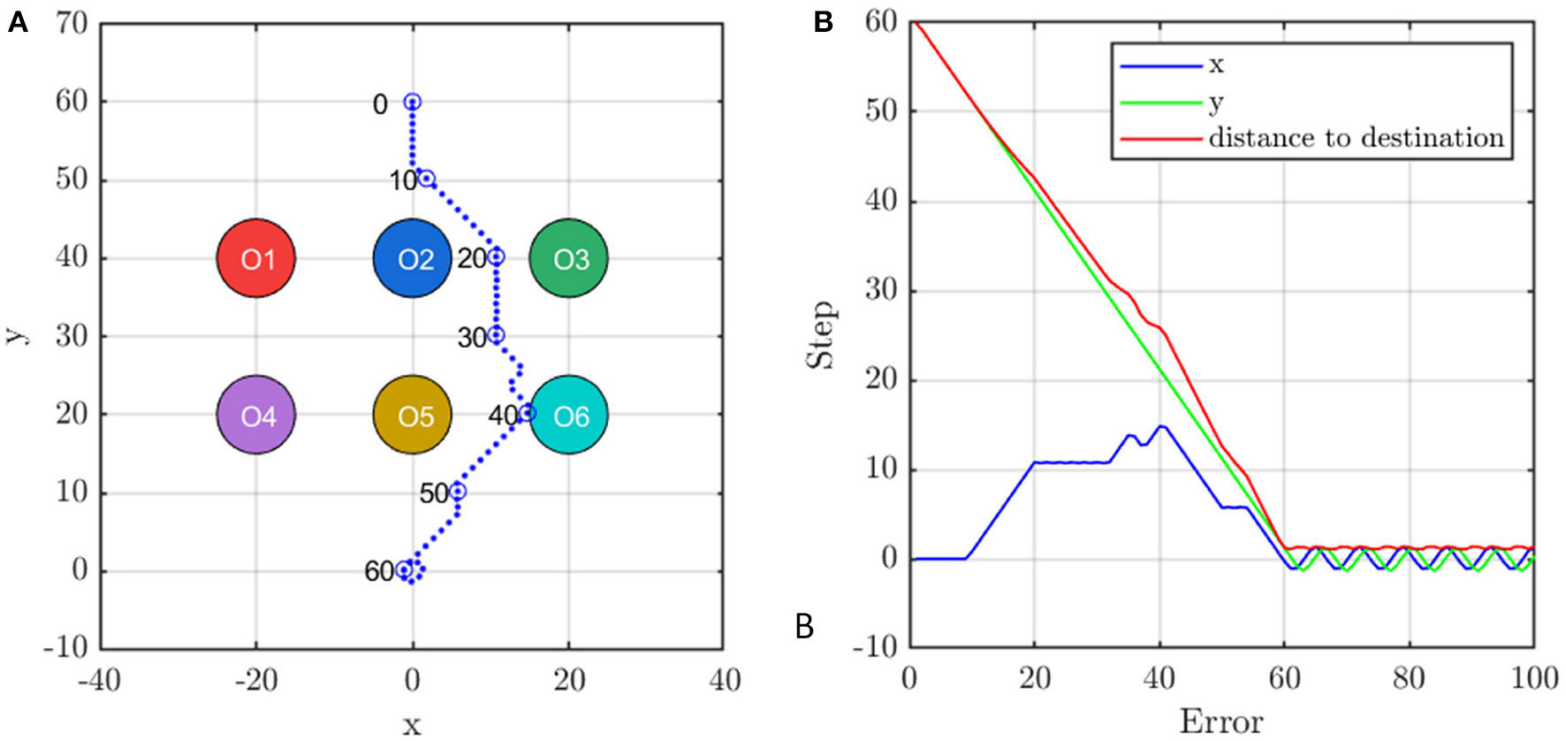
The agent's **(A)** trajectory and **(B)** the distance to the destination during avoiding obstacles. The six colored circles are the collision zone of obstacles and the blue dots are the trace of the agent.

To show how the proposed multi-agent formation control method avoids multiple obstacles, the scenario as shown in Figure 11A is presented, where six static obstacles locate in point [−20, 40], [0, 40], [20, 40], [−20, 20], [0, 20], and [20, 20] and the agent is initialized in [0, 60] with velocity [0, 0]. The target is [0, 0]. The blue dots represent the trajectory of the agent which is remarked by blue circles every 10 control steps. The collision zone of obstacles are represented by six colored circles.

In the beginning, the agent moves downward but turns right at the 10th control step to avoid Obstacle 2. At control step 20, the agent corrects its direction to approach target position. However, it must change direction at the 30th control step to avoid Obstacle 5. Finally, the agent faces the target again at the 40th control step and becomes stable at the target position after 60 steps. Figure 11B records the deviation of the agent from the target position. It is observed that the agent always approaches the target in *y*-direction but adjusts its velocity in *x*-direction to avoid the obstacles which are faster than any policy that changes the vertical velocity.

Next, we illustrate how the agent uses the unit controller to avoid multiple obstacles by fusion. By fusing the pair-wise state-action value of the six obstacles using equation (18), the agent can respond to the obstacle, that has a bigger threat, with a higher priority and thus, avoids more than one obstacle in the environment. To testify that the min-max fusion method indeed guides the agent responding to the most likely threat, we compare the min-max state-action value with two other kinds of most likely threat, i.e., minimum distance threat and minimum left time threat.

**Figure 12:**
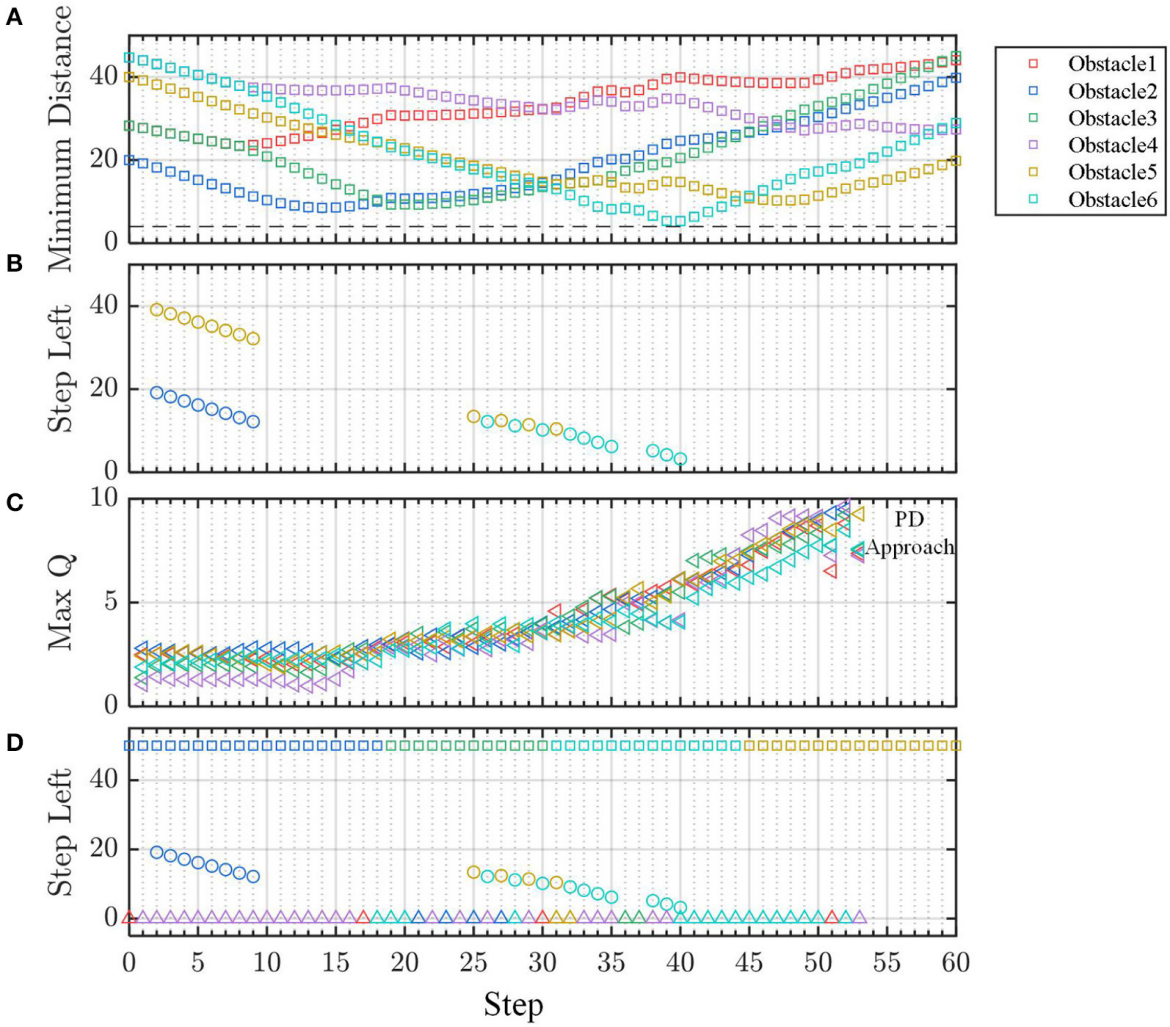
The **(A)** distance, **(B)** prediction of left steps, **(C)** *Q* value of the fused policy for obstacles. **(D)** Comparison of three kinds of threat prediction.

The minimum distance threat comes from the nearest obstacle, as shown in Figure 12A. However, the nearest obstacle may not have the biggest threat because the agent may move far away from this obstacle. Thus, the direction of the motion needs to be considered.

Supposed that the agent keeps moving at current speed in a straight line, it will reach the position which is the nearest point *M* (as shown in Figure 1C) to the obstacles in the straight line. Let **d**_*m*_ be the minimum distance to the obstacle in the straight line and Δ*t* be the left time for the agent moving from the current position to point *M*, we have


(21) $$\begin{array}{r} {\text{d}}_{m}=\rho_{O}-\frac{{\overset{∙}{\rho }}_{O}\left(\rho_{O}\cdot {\overset{∙}{\rho }}_{O}\right)}{|{\overset{∙}{\rho }}_{O}{|}^{2}} \end{array}$$

(22) $$\begin{array}{r} \Delta t=\frac{\rho_{O}\cdot {\overset{∙}{\rho }}_{O}}{|{\overset{∙}{\rho }}_{O}{|}^{2}} \end{array}$$

For all the obstacles, whose minimum distances **d**_*m*_ are less than the safe distance (|**d**_*m*_| < *d*_safe_), the biggest threat to the agent comes from the obstacle, for which it has the minimum left time Δ*t* to the point *M*. This is the second most likely threat criterion called minimum left time threat. According to equation (23), the left time (equivalent to left steps) for all the obstacles is shown in Figure 12B. In a similar way, according to equation (19), the maximum state-action value *Q* for the obstacle is shown in Figure 12C.

The aforementioned three kinds of threats are predicted and compared in Figure 12D. It is observed that the most likely threat indicated by minimum-maximum *Q* value is consistent with that of minimum left time threat in most of the steps, which validates our multi-agent formation control method.

#### 4.2.2. Demo: Multi-Agent Line Formation

Line formation is one of the most common formations in MAS. However, many formation control methods may be unreliable the line formation because they do not consider collision avoidance (Li et al., 2019a; Guo et al., 2020). The controller trained by static obstacles cannot ensure that the agent successfully avoids the moving obstacles. However, in some engineering scenarios, taking the other agents in the formation as static is reasonable. On the one hand, without filtering technique, the estimation of other agents' velocity may be unusable due to the observation noise. On the other hand, in most cases, like ground robots and quadrotors, the safe distance *d*_*safe*_ between agents is much larger than the agent's moving distance Δ*d* within decision interval Δ*t*. When *d*_*safe*_≫Δ*d*, the dynamic obstacle can be approximated as static because the internal logic of the controller is that if command makes the agent go away from the obstacle, then it is good; otherwise, it needs to be adjusted. In other words, the agent has enough time to find what works by trial and error, which is the advantage of the controller by learning. In the last demo, we present a line formation control scenario for four agents. Each agent is equipped with the DQN controller trained in section 4.1.1. We designate agent 1 as the leader and agent 2, 3, and 4 as followers. The expected target of the four agents are set as [0, 0], [0, 20], [0, −20], and [0, −40], respectively. In addition, their initial positions are set as [0, 0], [0, 20], [0, −20], and [0, −40] respectively, and their initial velocities are all zeros.

**Figure 13:**
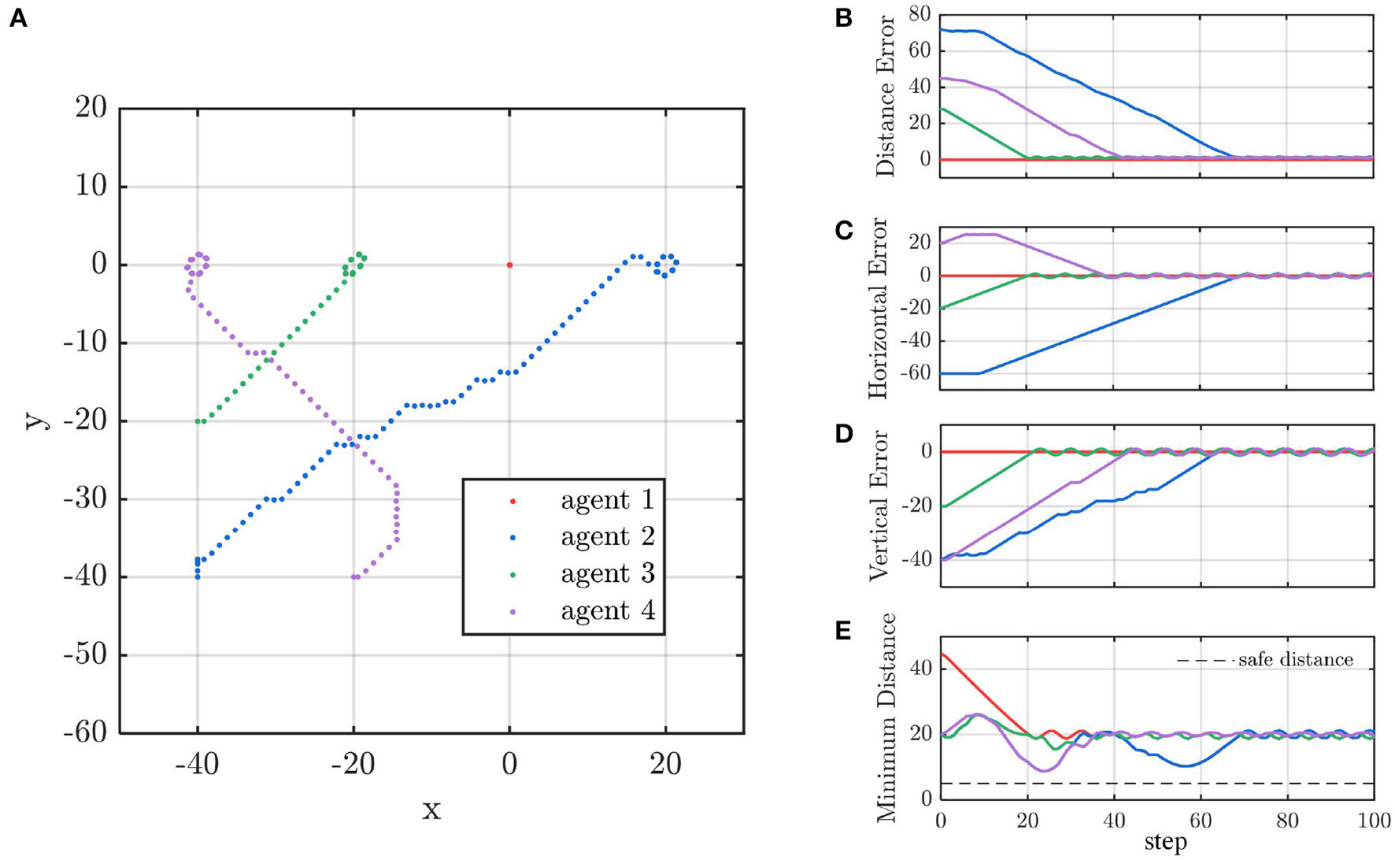
**(A)** The agent's trajectory. **(B–D)** The agent's distance to destination and **(E)** the distance between agents.

The trajectory of the agents is shown in Figure 13A. Agent 3 moves toward to its target position because there is no obstacle in its way. To avoid agent 2, agent 4 turns right and then turns back. Accordingly, agent 2 does not aim at its target position at the beginning to avoid collision with agent 4. Finally, the four agents form a linear formation. Figures 13B–D indicate that the distance between any two agents is more than the safe distance *d*_safe_ = 5, which validates the safety of our control method. As shown in Figure 13E, agent 2 changes its vertical speed instead of horizontal speed to avoid a collision. In the contrast, agent 4 adjusts its horizontal but not vertical speed.

## 5. Conclusion

Aiming at the problem of potential collision among agents in multi-agent formation control, an intelligent decomposition and fusion formation control method is proposed in this article. The multi-agent formation control is decomposed to the pair-wise unit formation control method where only one obstacle is considered. Then, the DQN controller for unit formation is trained following our episode mechanism design and reward shaping. Finally, by min-max fusion of all the pair-wise state-action values, the agent can first respond to the most likely threat among multiple obstacles without extra training. The demo of action field and unit formation control validates our unit formation DQN controller. The simulation results of avoiding obstacles and line formation show that our control method based on deep reinforcement learning can realize multi-agent formation with collision avoidance. In the future, obstacles with high dynamics can be taken into account, and the reward function can include optimal conditions like minimizing energy, and the fusion method also can be trained by the reinforcement learning method.

## Data Availability Statement

The original contributions presented in the study are included in the article/supplementary material, further inquiries can be directed to the corresponding author.

## Author Contributions

NX did research progress, simulation, and result in analysis and wrote the original draft with YH. LC supervised the work and revised this article. All authors contributed to the article and approved the submitted version.

## Conflict of Interest

The authors declare that the research was conducted in the absence of any commercial or financial relationships that could be construed as a potential conflict of interest.

## Publisher's Note

All claims expressed in this article are solely those of the authors and do not necessarily represent those of their affiliated organizations, or those of the publisher, the editors and the reviewers. Any product that may be evaluated in this article, or claim that may be made by its manufacturer, is not guaranteed or endorsed by the publisher.
